# The treatment of alcoholism that should not exist: Addiction, East German doctors, and Western methods in the German Democratic Republic

**DOI:** 10.1017/mdh.2025.9

**Published:** 2025-10

**Authors:** Markus Wahl

**Affiliations:** Lehrstuhl für Geschichte der Medizin, Friedrich-Alexander-Universität Erlangen-Nürnberg, Erlangen, Germany

**Keywords:** Alcoholism, Transnational, Knowledge transfer, GDR History, Treating addictions, Iron Curtain

## Abstract

Addiction was considered ‘alien to Socialism’. At least, that was the narrative upheld by the socialist East German state, which thus followed the traditional argumentation of socialist and social democratic movements since the turn of the century. While the state clung to this ideological claim, the consumption and abuse of beer, spirits, and benzodiazepines continued to increase. However, there was never a central strategy for the treatment and prevention of addiction in the German Democratic Republic (GDR). The hesitation and ignorance of the state authorities created a vacuum that was filled by local initiatives and expert discussions aimed at improving the situation of people with addictions. In this article, I analyse the introduction of new treatment methods in a psychiatric hospital in the GDR and show that doctors, psychologists, patients, and local officials had certain freedoms to test new approaches, many of which originated in the West. Even though they had to adapt concepts such as the ‘therapeutic communities’ of British reformer Maxwell Jones to the specific socialist and East German context to avoid restrictions by state authorities, the Berlin Wall did not prevent the transfer of knowledge. This article, therefore, paints a nuanced picture of the therapeutic methods used to treat people with addiction in the GDR. From condemning individuals as outcasts of socialist society for socially deviant drinking behaviour and relying exclusively on aversion therapy and moral accusations, there was a shift towards a mixture of treatments that became increasingly specialised and individualised, especially in the 1970s and 1980s, comparable to Western standards.

## Introduction

‘We do well if we look for solutions ourselves and do not wait for “miracles from outside”’.^1^ This quote from Erik Winter at a workshop on addictions in 1987 does not refer to solutions from other countries. Instead, as one of the leading East German doctors for alcohol disorders, he was indirectly criticising state departments. Leading party officials were still struggling to address – and provide a central strategy against – addictions by the late 1980s. Since the founding of the German Democratic Republic (GDR) in 1949, the state had claimed, ‘In our workers’ and peasants’ state, people do not need alcohol to be happy’.^2^ The initial enthusiasm of the socialist vanguard went as far as expecting that, like prostitution and begging, alcoholism would vanish and become a phenomenon of the past owing to the social transformation toward socialism.^3^ Subsequently, addiction developed into a highly politically and ideologically relevant topic for the GDR, similar to the issues of suicides and infant mortality. They represented indicators of the success of the socialist project in comparison with capitalist states. Addiction and abuse thus became a vehicle for propaganda against the ‘decadent’ and ‘exploitative’ West, where people supposedly needed alcohol to stay happy and to forget their worries. With constantly repeated slogans such as ‘addiction is alien to socialism’, state officials not only ignored and denied an emerging and worsening social problem as a possible outcome of socialist society but also individualised a developing addiction as the fault and failure of the person.^4^

By externalising the issue as a relic of the past, it appears that the medical discussion of, for example, recognising alcohol dependency as a disease had not reached the upper echelons. However, this is only partly true, as internal discussions within the ministries reveal. Instead, the state’s ideological narrative caused a dead end that its representatives could not overcome due to fear of Western media and an uproar among their population. Here, the memory of the upheaval from 17 June 1953, as well as the failed attempts to limit the sale of alcohol near and in workplaces during the 1950s, hampered any progress in tackling this social issue publicly.^5^ As late as 1979, the medical director of the People’s Police Hospital in Berlin wrote in his remarks for the Health Ministry that ‘[t]he drinking habits have remained the same, only the occasions have changed’.^6^ Alcohol played a vital role in people’s everyday lives in the GDR: as an unofficial currency for informal exchanges of labour and material, and as a constant companion at any work or leisure events, always available on a shelf in the supermarket, which was never empty.^7^ The historian Thomas Kochan concludes that the GDR was an ‘alcohol-centred society’, which the drastic increase in official statistics of alcohol consumption per capita proves.^8^ By 1988, the East Germans were even the world champions in drinking schnapps – a phenomenon that was based on the history of drinking and distilling alcohol in this area.^9^

In this alcohol-centric atmosphere, state officials resigned themselves to focusing instead on the criminal perspective of alcohol abuse. According to internal statistics, over seventy per cent of registered offences of state slandering occurred under the influence of alcohol and – more dangerously for the GDR and subsequently for the person loudly criticising the state – mostly in public places, especially pubs.^10^ This focus on criminal behaviour enabled state officials to condemn the issue of alcohol abuse and addiction as a singular problem of a few.^11^ According to their reasoning, these people had consciously decided against integrating into the socialist society and thus had to face harsh sanctions.

For instance, the GDR passed a regulation in 1961 allowing charges to be levied on people whom police or ambulances had to pick up due to their drunkenness. In a state where health care and provision were propagated to be free and generally accessible, charging high fees was a drastic punishment. The state rationalised this measure initially by claiming that the state budget ‘must not be used and wasted frivolously and uselessly by unscrupulous individuals’.^12^ After another increase in fees in 1984, the newspaper *Berliner Zeitung* applauded this step and still argued that this regulation ‘is not so much directed against the abuse of alcohol, which is morally condemnable, but against the abuse of social institutions by drunks’.^13^ A basic eugenic and moralising attitude is evident in both statements. They regarded people with alcohol addiction as inferior humans who deliberately withheld their labour from society. Therefore, they did not deserve the achievements of state socialism. The law was drafted with this in mind and focused exclusively on punishment, without offering medical or social help to those affected.

Winter’s statement at the beginning of this paper criticised the briefly outlined general situation that had hardly changed by the end of the 1980s. The last decade of the GDR saw some relaxation towards TV shows addressing alcohol abuse and the publication of books, for example, about the experiences and struggles of a woman with alcohol addiction in a socialist state.^14^ However, as in many other cases, such as psychotherapy, the real change happened at the local level.^15^ The state’s struggle, refusal, and ignorance left a vacuum in this social issue, which doctors like Winter, psychologists, and patients filled with their own initiatives. This unexpected leeway enabled them to discuss and, ultimately, implement ideas to improve the situation of people with addictions in their communities. Subsequently, it remained a decentralised effort by individuals at the local level in a supposedly centralised state until the end of the GDR in 1989/90.

The search for solutions for the therapy and care of people with addictions, which Winter referred to in his statement, was not disconnected from the rest of the world. Moreover, the reforms and new methods were not invented by those who assembled at the mentioned conference in 1987. This article, therefore, discusses the transfer of selected ideas, concepts, and therapy methods within the Eastern Bloc and with the West, using the example of alcohol addiction during the forty years of the GDR. The research question is how the participation of East German experts in international debates on the treatment of addicts can be traced at the local level. To investigate this transfer of knowledge, I utilise different types of primary sources. These include published conference proceedings and journal articles, as well as unpublished sources such as internal documents from ministries held in the *Bundesarchiv* (Federal Archive – BArch) in Berlin and patient files from the District Hospital for Psychiatry and Neurology in Arnsdorf near Dresden. Arnsdorf was the largest psychiatric hospital for the care of mentally ill and addicted people in the GDR and the leading institution for the district of Dresden.^16^ It is thus exemplary of the social and medical treatment of alcoholics locally, often far removed from state policy and expert discussions. The case study of Arnsdorf and the doctors’ international connections demonstrates that the state indirectly welcomed and relied on this transfer of knowledge and the implementation of local solutions, despite its overall refusal and apparent ignorance.

## Stalinism, Pavlovism, and international exchange during the 1950s and 1960s

Ivan Pavlov (1849–1936) and his experiments on conditioning and research into the nervous system became the foundation of Soviet and socialist human sciences. After the Second World War, the Soviet and socialist governments and experts in the 1950s attempted to introduce Pavlovism on a broad scale in neurology and psychiatry, including in the GDR after its foundation in 1949.^17^ Ultimately, the aim was to demarcate Soviet human sciences from the West and Freudian psychoanalysis through alternative approaches, thereby feeding into the old dispute between endogenous and exogenous causes of deviant developments and behaviours. However, as a result of this competition triggered by the Cold War, Pavlovism developed into an overstretched concept, partly to the detriment of patients. One example is Pavlovian sleep therapy developed by Dietfried Müller-Hegemann (1910–1989), a GDR psychiatrist and member of the ‘State Pavlov Commission’. This consisted of pharmaceutically induced and prolonged sleep in conjunction with a psychotherapeutic programme over a period of two to six weeks. Not least because this therapy had not proven effective in practice and ended fatally for a high-ranking party member, the therapies based on Pavlov quickly disappeared again by the mid-1960s.^18^ As the medical historian Matthias Hoheisel notes, only the ‘conditioned reflex’ remained and reappeared in scientific discussions in the GDR. As a replacement for Pavlovism and Soviet authors, the reports and works of the World Health Organisation (WHO) became one of the primary ‘Western’ references in studies from the 1960s.^19^

A similar trend is seen in the discussion around ‘alcoholism’ in East German scientific journals. However, only a few members of the medical community published on addiction during the 1950s. Experts like the aforementioned Müller-Hegemann focused on and addressed addiction as a result of societal conditions, supporting the arguments of the socialist party against capitalism.^20^ Others investigated the effects of aversion therapy, using disulfiram or apomorphine to create a ‘conditioned reflex’ in the affected people towards alcohol.^21^ The application of these drugs to create discomfort, high blood pressure, nausea, and dizziness in patients while they drank different amounts of alcohol reached its international peak in the 1950s and early 1960s. At the time, the medical community celebrated them as the long-awaited cure for alcohol addiction.^22^ The idea of conditioning people to refrain from what was classified as deviant behaviour was popular, and doctors in the United Kingdom and Czechoslovakia, for example, introduced this method as a standard therapy for homosexuality as well.^23^ However, the inhuman nature of aversion therapy led to a rapid decrease in its use internationally by the mid-1960s.

At the state level and in the public sphere, discussions on alcohol abuse were rare and largely focused on reducing criminality and correcting socially deviant behaviour.^24^ Only the exhibitions at the German Hygiene Museum in Dresden, which was founded in 1912 and which has been responsible for educating the public about hygiene and health ever since,^25^ and some advice literature warned the general public of the medical and social dangers of alcohol.^26^ Internal debates within GDR ministries also draw a different image – not least due to the renewed steady increase of alcohol consumption since 1945.^27^ Members of the Health Ministry developed extensive draft proposals during the 1950s on how to tackle alcohol addiction and how to control and treat affected people. These suggested regulations and laws consisted of wide-ranging reforms, which included dedicated counselling centres for people with addictions, which already existed for other chronic illnesses such as diabetes. Therefore, they also strongly focused on help and care alongside the known sanctions. With their drafts, the members of the Health Ministry thus tried to follow international reforms and progressive ideas while acknowledging alcoholism as an illness and social issue.^28^ This potential official recognition might have been why the Council of Ministers and Health Minister Max Sefrin (1913–2000) rejected the proposals and instead recommended establishing an ‘Alcoholics Prophylactic Centre [*Trinkerprophylaktorium*]’ in Berlin as a test run.^29^ This stalling resulted in the ministries never elaborating further on these proposed regulations.

Despite these apparent limitations during the early decades, some East German doctors were already involved in international debates. The psychiatrist Hugo von Keyserlingk (1909–1980) was – like his son of the same name (b. 1934) after him – internationally well connected and an expert in the field of addictions. In his articles and books, he offered a detailed analysis of the increasing alcohol intake in East Germany after the Second World War. Unlike Müller-Hegemann, he did not claim that addiction would be a relic of past society. Instead, he pointed to causes within the socialist society while citing international studies and their observations and proposed solutions at the time.^30^ This open discussion about the shortcomings of the socialist society and state in East German scientific journals may come as a surprise, but it is important to remember that these were expert-level discussions that only reached a specific audience and hardly a wider public – and that their articles were therefore not subjected to rigorous scrutiny.

East German doctors also strove to expand their participation in international developments and tried to convince the state of their benefits. One example is Werner Schmincke (1920–2003), a well-known GDR social hygienist in Dresden and member of the ‘Doctors’ Commission at the Central Committee’. In June 1958, he proposed the establishment of a ‘National Committee for Researching Alcoholism in the GDR’ to the Ministry of Health on the occasion of a conference on alcoholism organised by the Ministry itself in Dresden that November. In his opinion, this new committee should join the ‘International Bureau Against Alcoholism’ [later renamed the International Council on Alcohol and Addiction (ICAA)], an organisation based in Lausanne, Switzerland. He argued that a national committee would face less resistance from capitalist states, that is, West Germany, to become a member of this supranational organisation than an institution like the German Hygiene Museum in Dresden. At a time when the GDR actively sought international recognition and during the accelerating Cold War, Schmincke’s suggestions should have fallen on fertile ground. To support his idea, he reported that the executive director of the ICAA visited the GDR in April 1958: ‘He is English, and it emerged in conversation that he is committed to the progressive direction of the Labour Party’.^31^ According to Schmincke, this man strongly supported the GDR’s application to become a member. Subsequently, state officials apparently encouraged the request, mainly due to this organisation’s close ties to the WHO.

The famous visitor from the United Kingdom was Archer Tongue (1919–2006), the executive director of the ICAA from 1952 to 1990. According to an obituary, it was only through him that this organisation become an important international player and a platform for debates on alcohol addiction across political boundaries. He was a family man, highly educated and diplomatic, with a ‘friendly demeanour and […] quiet persistence’, and often welcomed international experts at home.^32^ These qualities and connections led to him repeatedly appearing as an author in East German scientific journals and as a guest at conferences in the GDR from the end of the 1950s onwards.

However, this internationality had its limits. It is not clear whether Schmincke’s committee materialised, but it did not join the ICAA. Instead, the GDR sent its representatives from the ‘Society for Forensic Medicine’ to this organisation, a move in line with the state’s narrative and focus on alcohol and criminality.^33^ Nevertheless, other selected East German doctors could participate in the international seminars organised by the ICAA. Rudolf Neubert (1898–1992), a social hygienist and author of crucial popular advice literature since the 1920s, for example, took part in the second summer course in Geneva in 1956 and the twelfth meeting in Prague in 1966. This gave him, as an esteemed expert, continued access to research, scientific literature, and reform ideas for the treatment of alcohol addiction worldwide.^34^ On this international stage, East German doctors always claimed that, in contrast to capitalist countries and due to the societal transformation in the socialist states, alcohol and addiction would cease to be an issue. This standard phrase appeared in many preambles to laws and public statements and was especially necessary for the GDR’s representation on the international parquet.^35^ Therefore, doctors who travelled abroad had to include this declaration out of conviction or necessity in order not to lose their chances as ‘travel cadres’ to the West.

One important example was Rolf Thränhardt (b. 1918), who was the scientific director of the German Hygiene Museum in Dresden in the 1950s and later head of the Department of Health Education in the GDR Ministry of Health.^36^ He belonged to the international scientific community from the very beginning and, like Neubert, took part in the ICAA conference in Prague in 1966. A person in this high position could be expected to adhere to party doctrine as a loyal or opportunistic follower. It is irrelevant which case applies to him, but it is crucial that his work, his reputation, and his social position lent him credibility. In his report to the GDR Ministry of Health on the papers and discussions at the Prague conference, Thränhardt also addressed the current situation in the GDR – and did not hold back with criticism:

The echoless course of the Dresden Alcoholism Conference of 1958, the deafness of the state to the warnings of the medical profession, and grotesque anachronistic usages such as the degrading distribution of deputised brandy to some professional groups [e.g. miners, M.W.], apparently intended as a stimulant, with the psychologically inevitable consequence of a consumer attitude, characterise the situation.^37^

In this harsh statement, Thränhardt referred to the 1958 conference, during which Schmincke had proposed founding the ‘National Committee on Researching Alcoholism’. According to him, their discussions and findings had no impact on legislation. Instead, the government only managed to introduce a regulation to charge intoxicated people for being transported by ambulances or police.^38^ A ban on the still-common practice of deputised alcohol (*Deputatalkohol*), a traditional form of additional monthly compensation for miners for their hard work by the local distilleries with high-proof schnapps, which Thränhardt criticised in his statement, did not follow until the end of the 1960s:

It is banally simplistic to speak of poverty or affluence alcoholism. Drinkers are often socially valuable people outside of drunkenness. American corporations have apparently recognised this better than the moralising opinion prevailing in this country!^39^

With this report, he dealt a heavy blow to the state narrative and ideological framework of the time. First, he rejected the common notion that alcohol addiction was due to poverty or decadence, simultaneously refuting the ‘relic of capitalism’ theory of the socialist vanguard. Secondly, his statement that American companies would pay for the therapy of their valued workers was bound to be a thorn in the side of the socialist party, especially because he argued that the ‘class enemy’ with its ‘capitalists’ cared more about its employees than the proclaimed humanistic and egalitarian society in the GDR. Thirdly, he pointed out the still moralising attitudes toward alcohol and alcohol addiction – especially among functionaries, but also in the medical profession – which needed to be overcome. Thränhardt’s criticism did not have any impact on his career, as it was an informal and internal report, and he was held in high regard both in the GDR and internationally. However, no notable change in state policies toward alcohol addiction was made after his outburst.

In contrast to the state level, the scientific discussions in the GDR and the international exchanges appeared to continue unhindered – even after the final border closure between East and West Germany in 1961. For example, the new classification and description of alcohol addiction by the famous US alcohol researcher E. Morton Jellinek (1890–1963), who was also fluent in German, already became the new standard at the expert level.^40^ With the differentiation of various forms and manifestations of alcohol addiction, his research and categories were one vital step towards more individualised treatment strategies. Thränhardt also positively referenced Jellinek in his report to the ministry in 1966. He further embraced the ‘bifocal therapy’ – meaning the combination of pharmaceutical therapy with psychotherapy – put forward by the French expert R. Reyss-Brion. According to Thränhardt, psychotherapy, especially in the form of group therapy, was favoured by many participants at the ICAA conference, and the American psychiatrist Hoff urged to make psychotherapy the primary method in the treatment of people with alcohol addiction.^41^ Therefore, the international medical community abandoned the enthusiastic use of disulfiram and apomorphine and focused on reforms and individual therapeutic plans instead of standard procedures.^42^

Apart from these positive mentions of ‘capitalist’ ideas, Thränhardt made one significant qualification to his report. For him, the therapeutic concepts of another participant at the 1966 conference, Jaroslav Skála (1916–2007), would be better suited to the GDR context. Skála was also an important figure in the reform of alcohol addiction treatments, and his Apolinar clinic in Prague was internationally renowned.^43^ This limitation seems to be a tendency to follow socialist brother states. Nevertheless, Skála’s concepts were partly based on experiences from other countries, and his developed system of registering cases, setting up medically or psychologically run therapeutic clubs for patients, and specialised clinics was internationally recognised at the time.^44^ According to historian Adéla Gjuričová, his status in Czechoslovakia (CSSR) and the world allowed him to try out various approaches that were often subversive for the socialist state.^45^ This observation also applies to the aforementioned East German experts on the international stage. It further refutes the state’s attempts and claims to free the GDR of Western influences during the 1960s. On the contrary, there was a well-established transfer and openness towards East and West therapeutic concepts in the 1960s, as shown by many recent research projects.

## International connections and outdated methods in Arnsdorf during the 1960s

The international transfer of knowledge also took place within the walls of the District Hospital for Psychiatry and Neurology in Arnsdorf near Dresden. Therefore, this hospital is used as a case study in this section to illustrate the gap between the experts’ discourse and the local implementation of therapeutic methods. To contextualise the findings, I will include references and comparisons with the situation in the Brandenburg District Psychiatric Hospital.

In June 1968, doctors from the GDR, CSSR and Austria gathered at the ‘Scientific Workshop on Fundamental Issues in the Treatment of Alcoholics’ in the Arnsdorf hospital to discuss their different approaches for treating people with alcohol addiction and their ‘success’ rates.^46^ One of the participants was the well-known psychiatrist Ehrig Lange (1921–2009), a local representative of the Ministry of Health and professor at the Carl Gustav Carus Medical Academy in Dresden, who had apparently organised this international convention. Lange himself is a case in point for the expertise transfer across the Iron Curtain. In his work, he openly advocated and followed the British concept of an ‘open-door system’ to remove the ‘prison-like character’ and overcome the ‘old’ reputation of the psychiatric hospital.^47^

According to his summary in the journal *Psychiatrie, Neurologie und medizinische Psychologie*, Lange invited Kornelius Kryspin-Exner (1926–1985), an Austrian psychiatrist whose name has been recently linked to accusations against the psychiatric hospital in Vienna.^48^ In this institute, he and his colleagues applied harsh treatments to assumed ‘deviant’ children during the 1960s.^49^ At the time of the conference, Kryspin-Exner had just published about the methods used at the ‘Open Institution for Alcoholics in Vienna-Kalksburg’ in 1967.^50^ He also reported on this facility during the meeting in Arnsdorf, claiming that they would adjust therapy methods and duration according to the individual case and refute any uniform and forceful interventions. Most of his patients would receive disulfiram, while the application of apomorphine would be strictly limited. This statement indicates the increasing departure from aversion therapy towards psychotherapeutic methods internationally, as Thränhardt reported about the ICAA conference two years earlier.^51^ Skála also participated and gave an overview of the rehabilitation of people with alcohol addiction in Prague and its development since 1945.^52^ Even though only one participant came from a capitalist state, they were all leading experts and reformers in treating alcohol addiction at the time.

Karl-Heinz Wieder (1920–2001), the medical director of the psychiatric hospital in Arnsdorf between 1965 and 1987, and his contributions at the conference provide this article with insight into the divergences of postulates and experienced reality. His statements contradicted the situation and mentality in his institution at the end of the 1960s. In his presentation, Wieder claimed that individual and group psychotherapy would be favoured in his hospital over the application of aversion therapy – but the latter is regarded as ‘indispensable’ for rehabilitating a person with alcohol addiction.^53^ A ‘patient club’ would also exist and play an essential part in the aftercare. In this way, he aligned himself with the international experts and their reformed treatment strategies.

However, Wieder indirectly demonstrates a significant limitation: the described discussions and transfers of ideas at the macro level of medicine often did not reflect the situation on-site. In the patient files that I was able to access from this institution, there was never any mention of a ‘patient club’ in the 1960s.^54^ The reason for this could be that the medical staff made no reference to this aftercare opportunity in this sample. Other methodological issues could lie in their lack of knowledge or time pressure, which led to missing information in the patient files. Nonetheless, ‘patient clubs’ or ‘therapeutic clubs for abstinent living alcoholics’ became a widespread phenomenon and an important pillar of local support in the GDR by the 1970s. According to the chairman of the Dresden club at the 1979 Brandenburg conference, they had only founded this self-help group in 1978.^55^ Nevertheless, Wieder might have referred to a different group or form of support, appropriated the terminology from Skála for prestige purposes, or meant the ‘Evangelic Working Group for Averting the Danger of Addiction’ (AGAS). The latter was the indirect successor of the Blue Cross, which was banned in East Germany after 1945. The AGAS was founded in Dresden in 1960 and offered support and events for the affected people and their relatives within the framework of religion.^56^ Until the middle of the 1970s, AGAS and other church groups were the only comprehensive outpatient and aftercare opportunities for people with addictions.

This situation and the slow recognition of alcohol dependence as an illness impacted the terminology used for those affected and the therapeutic methods, contradicting Wieder’s statements at the international conference in 1968. In the 1960s, the doctors in Arnsdorf still used the old diagnoses such as ‘alcoholism [*Trunksucht*]’ or ‘chronic alcoholism in the addiction phase [*chronischer Alkoholismus in der Suchtphase*]’.^57^ Jellinek and his classifications were apparently neither used nor known to the medical staff. One example is Herbert,^58^ a 24-year-old sewer cleaner who was admitted to Arnsdorf in 1968 with the diagnosis ‘chronic alcoholism in a primitive personality’. His ‘withdrawal treatment’ was part of his conviction for drunk driving without a licence after a five-month prison sentence in 1967. The court justified the verdict that ‘[t]he causes of the crime do not lie in alcohol but in the fact that the defendant for many years did not muster the will to behave correctly in our social order as it is required’.^59^ Written a year after the Prague conference, the moralising attitude that Thränhardt had criticised and which was part of the GDR’s social engineering project to create socialist personalities through ‘Instruction, Information, Education, Re-education’ becomes visible.^60^ The predisposition described was widespread among the medical staff in Arnsdorf due to their age and socialisation. Many doctors and nurses who worked in this and other medical institutions in the GDR in the 1950s and 1960s had completed their training in the Weimar Republic or the Third Reich, which had a profound effect on the medical and social treatment of their patients.^61^

This situation is also reflected in Herbert’s treatment, as he and others in the 1960s – contrary to Wieder’s statements – only received work and aversion therapy. The latter was still justified through the Pavlovian concept of the ‘conditioned reflex’ in the form of the so-called ‘alcohol test [*Probetrunk*]’ – a euphemism for aversion therapy – which served as a deterrent. Doctors applied it, taking into account medical indications and following their subjective judgment of a person. This led to different numbers of alcohol tests being carried out depending on the patient’s age, gender, social background, and behaviour.^62^ Aversion therapy thus fitted in with the moralising attitude of the medical staff towards patients with alcohol addiction, which often prevented an individualised treatment plan. Herbert’s discharge letter also mentions psychotherapy, but this did not appear in the medical progress sheets or other documents in his patient file. Compared with other discharge letters, these were often only standardised writings with some changes, e.g. regarding the recommended medication after discharge.^63^

Nonetheless, Wieder also mentioned aspects in his presentation that can be verified with the sample of patient files.^64^ For example, doctors tried to cooperate with relatives and companies to prepare for the discharge and reintegration of their patients. The aim was to establish a network of social support and social control. In many cases, as with Herbert, they would even enquire with the company about the situation of their former patients after a year or two.^65^ These methods reveal the paternalistic approach that characterised GDR society. It also meant that people with alcohol addiction could not choose to hide their illness from their social environment. On the contrary, the diagnosis code 303 for alcohol dependency was noted on their social insurance card, which they had to hand over to their employer. This situation yielded both positive and negative effects. On the one hand, they may have received support in their rehabilitation process, and an appointed colleague or supervisor took care of them and monitored their use of disulfiram. On the other hand, the confrontation in which superiors controlled their employees’ medication could also lead to social conflicts in this hierarchical environment. Moreover, the disclosure of this illness often resulted in stigmatisation, ignorance, and deliberate provocations by colleagues with alcoholic drinks to test the patient’s abstinence.^66^

Another aspect Wieder referred to in his paper in 1968 was an educational system that nurses had introduced so that patients who violated the house rules were given points.^67^ The ramifications of this point system are unknown, but a patient’s refusal to take part in the important 1 May celebrations was still regarded as a negative personality change by the medical staff in 1971.^68^ Nevertheless, international developments in the treatment of alcohol addiction also influenced this institution, and group therapy became standard in Arnsdorf in the early 1970s – possibly as a result of the conference at this hospital in 1968. Another aspect was, as the authors of the commemorative publication on the one hundredth anniversary of Arnsdorf point out, that, although Wieder welcomed reforms in principle, he was described as a ‘man of small steps’. This characteristic could be the reason for the discrepancy between his statements at the 1968 conference and the slowly progressing reality in his own hospital.^69^

At the same time, a completely different situation prevailed in the addiction clinic of the Brandenburg District Mental Hospital with its head physician Hubertus Windischmann (b. 1931). He had been involved in the scientific debate since the 1960s with articles and conference papers, consulted Skála for a few months in Prague, and subsequently introduced psychotherapy and therapeutic clubs at his clinic in 1968.^70^ As a young doctor at the time, he belonged to a new generation that was to change the situation of people with alcohol addiction in the GDR over the last two decades.

In general, the 1950s and 1960s saw a constant struggle between the state taboo, the growing social problem of excessive alcohol consumption and alcohol addiction, and the reform ideas of experts in the East and West of the Iron Curtain. The transfer of knowledge appears to flow almost undisturbed by Cold War developments, like the final closure of the border between the GDR and the Federal Republic of Germany in 1961. The scientific dialogue continued to take place – albeit with some restrictions in place and only for selected ‘capitalist’ experts and East German ‘travel cadres’. The ICAA executive director, Tongue, had a key position in this transnational exchange and was an accepted official contact with the West by GDR state officials. Conversely, in his report on the ICAA conference in Prague in 1966, Thränhardt admitted to the Ministry of Health ‘that the work published in the GDR on the overall topic was only little known’.^71^ As other recent studies have shown, the transfer of knowledge often only flowed in one direction, from West to East. It suggests that Western experts were ignorant of the reform ideas originating in the GDR.^72^ Despite this observation, a development in the case of alcohol addiction gained momentum since the late 1960s, which brought with it an ever-increasing exchange of knowledge across the Iron Curtain and courageous measures by local doctors and psychologists to overcome a perceived stagnation in this area.

## Local initiatives, the State’s denial, and a global problem in the 1970s

As early as the 1960s, after turning away from Pavlovism, and increasingly in the 1970s, the understanding of medical therapy in the GDR changed. The view shifted from a purely biomedical approach to mental illness to an understanding of the person as a product of their socialisation and their social environment. While this view had always been considered in the social hygiene movement since the nineteenth century, psychiatrists often saw patients as objects that deviated from a defined ‘norm’. They were primarily interested in the science behind an illness and not in the person. East German doctors gathered at a conference in Rodewisch attempted to overcome this one-sided approach as early as 1963 with the ‘*Rodewischer Thesen*’.^73^ This work aimed to reform the prison-like character of the old psychiatric wards and to improve therapies and doctor–patient relationships. In the 1970s, its further development in the form of the ‘*Brandenburger Thesen*’, which was discussed at a conference in Brandenburg in 1975, as well as a generational change within the profession led to local initiatives seeking to improve the situations of alcohol-dependent people and undercut the trend towards alcohol dependence becoming a common phenomenon. In this section, I will show how an increased transfer of expertise between East and West and between doctors and patients, often in calculated defiance of state authorities, influenced the treatment methods and aftercare using the example of the hospitals in Arnsdorf and Brandenburg.

‘Once again, we are told to lock up our patients, some Vietnamese delegation is coming. We are not the police!’ According to a Ministry of State Security (MfS) report, doctors from the Charité complained about the admission procedures during international events in 1973.^74^ Two aspects of this statement are essential. First, despite the mentioned reform initiative from 1963, the psychiatric hospital still had the general reputation of being a prison-like and safeguarding institution for people deemed to be social deviants. In this case, the state abused this medical facility by ordering doctors to admit selected psychiatric patients to avoid public disturbances during diplomatic delegation visits or the 1973 ‘*Weltfestspiele* [World Festival]’. However, this procedure was often criticised and sometimes even rejected by the doctors in charge, as in the quote from the Charité.^75^ It also indicates that the aim of Lange and others to reform the psychiatric care system in the GDR towards ‘open-door’ institutions had not yet spread across the country. The reason was often the lack of material, financial, and human resources and outdated attitudes towards patients with stigmatised diseases, which hampered reforms in the entire GDR healthcare system.^76^

Secondly, the statement is based on a significant change: the new admission regulations for psychiatric hospitals from 1968. This law, in conjunction with a new penalty code, replaced the former procedure of forcibly committing people with socially deviant behaviour to psychiatric hospitals with the help of the police. From this point onwards, a district doctor could also issue a retroactive order in accordance with paragraph six for patients who were already in hospital if they or their relatives refused admission. In the name of the safety and health of patients and society, district doctors could impose this involuntary hospitalisation for up to six weeks before a court had to be involved, which was specified in paragraph eleven for longer or indefinite stays against a person’s will.^77^

This law was particularly important for people with alcohol addiction who had not accepted their condition. Out of the sample of 128 patients with this diagnosis from the hospital in Arnsdorf, 95 were in this facility after the new law was introduced in 1968. Nine of the individuals (9.47 per cent) were temporarily hospitalised against their will in accordance with paragraph six, and one person was admitted indefinitely according to paragraph eleven. Paragraph six was often used to acquire patients’ consent and understanding of their illness after withdrawal treatment with lectures, self-reflection as homework and group therapy, which was also practised similarly in other clinics and countries.^78^ To contextualise the Arnsdorf sample, a 1984 article by the GDR psychiatrists Helmuth F. Späte (1936–2017) and Harald Rogoll provides detailed analysis of the admissions to the District Hospital for Psychiatry and Neurology in Bernburg between 1969 and 1977. They concluded that 3.1 to 6.5 per cent of the annual hospitalisations were due to paragraph six, from which 10.5 per cent had a diagnosed alcohol addiction. With the argument that only two per cent of all admissions would turn into an indefinite stay under paragraph eleven, they celebrated – at least in the case of the Bernburg hospital – the apparently ‘human character of this law and its proven value in practice’.^79^

However, in a different article from the same year, the authors also pointed out that the actual implementation of this law varies depending on the interpretation of local doctors and courts. Their criticism went so far as to state that, on the one hand, many admissions were insufficiently justified, and the patient did not receive a written order or instructions regarding legal representation. On the other hand, the district doctors also failed to inform the courts, and unnamed but known authorities often requested the admission of patients to closed wards or for an indefinite period without a diagnosis to justify their order.^80^ While this article was published in a scientific journal that did not reach the general public, the outspoken defiance of experts exemplifies their leeway in the decades after Erich Honecker took over in 1971. It also illustrates a changed atmosphere, including ethical considerations and patient rights, in line with global developments.

This shift in attitudes towards psychiatry was visible in international debates regarding ‘deinstitutionalisation’ and ‘community care’. In the GDR, the second vital step towards improving care in psychiatric hospitals was the launch of the ‘*Brandenburg Thesen*’ – an East German version of the ‘therapeutic community’ developed by the British reformer Maxwell Jones (1907–1990). The historians Ekkehardt Kumbier and Kathleen Haack have analysed these theses, of which two versions exist: one from 1974 and one from 1976. In between was the conference in Brandenburg in 1975, at which the 1974 proposal by Siegfried Schirmer (1927–2013), Karl Müller (1922–1992) and Späte was discussed. After this conference, the second and final version was published in a depoliticised form, apparently under pressure from state authorities.^81^ According to Kumbier and Haack, the changes concerned criticism of the socialist society and its handling of people with psychiatric illnesses.^82^ Despite this ideological censorship, the authors of the 1976 version openly referred to many Western experts such as Erving Goffman (1922–1982) and Rudolf Karl Freudenberg (1908–1983) and the implementation of their theories in a socialist state.^83^ State censorship, therefore, did not hinder the knowledge transfer between East and West. As long as the GDR authors adhered to ideological conventions, they were able to propagate Western ideas in the East German scientific community.

In the case of Arnsdorf, the generational change by the 1970s also led to a shift in terminology and therapies. In 1975, at the time of the ‘*Brandenburger Thesen*’, the doctors at this clinic began to diagnose their patients using terms such as ‘*Chronischer Alkoholismus in der Suchtphase (Gamma-Trinker nach Jellinek)* [Chronic Alcoholism in the Addiction Phase (Gamma Drinker according to Jellinek)]’.^84^ As in section one, the old terminology ‘alcoholism’ persisted. However, the classification according to Jellinek, which had been the international standard since the 1960s, was included in brackets, indicating at least partial replacement. This mixture of the old and ‘not so new’ classifications also reflects the fact that the chief doctor was the same as in the 1960s, while he had a new assistant physician. In all patient files of the sample in which the name of the new assistant doctor appears, Jellinek was used as a reference.^85^ In other files, however, where different doctors were in charge of documenting the case, only the outdated and stigmatising terminology continued to be applied.^86^

The differing attitudes among the medical staff at this hospital and the change around the year 1975 are also visible in the treatment of people with alcohol addiction. As a result of the outdated terminology and views towards addicts, aversion therapy still played a crucial role in Arnsdorf until the mid-1970s, although this method was heavily criticised internationally and had already been largely abandoned. However, patients in this hospital, especially women, had to undergo up to six ‘alcohol tests’ during one stay.^87^ The gender-specific application was also connected to a senior doctor at this hospital, who used aversion therapy sessions to formulate moralising accusations against the patient. This attribution of blame for failures due to alcohol abuse, e.g. for neglecting their role as a mother, was apparently intended to increase the deterrent effect of the ‘alcohol tests’.^88^ After 1975 and with the promotion of the above-mentioned new assistant doctor to head of the ward, who was a woman, the number of ‘alcohol tests’ performed fell drastically to a maximum of two and already lost its moralising connotations.^89^ This quantitative analysis is striking and corresponds to the fact that the treatment methods depended heavily on the attitudes of those in charge. A case in point was the appointment of the new chief doctor, Hans-Dieter Koritsch (b. 1941), in 1980, who had previously worked at the Psychiatric Hospital Großschweidnitz. Under his supervision, the concept of aversion disappeared. Following Windischmann’s therapeutic approach in Brandenburg, he propagated the abandonment of ‘alcohol tests’ as a deterrent. This shift can also be understood as the final departure from Pavlovism and the ‘conditioned reflex’. Instead, he emphasised the advantages of disulfiram as a quick and early way of achieving and maintaining abstinence as a prerequisite for all other therapies.^90^

This generational change is also valid for other cities and clinics in the GDR. It had a significant impact on the attitudes and treatment concepts in psychiatric hospitals and towards alcohol addiction.^91^ Doctors and psychologists implemented their versions of Skála’s therapeutic clubs, opened day and night clinics to enable people with addiction to keep their jobs, and increasingly abandoned aversion and deterrence in their therapeutic catalogue, like in Arnsdorf during the 1970s.^92^ Until 1979, however, these efforts were highly localised and hardly connected – if at all, then through the personal acquaintance of the doctors. This year, the first conference on exchanging ideas about treating addictions through doctors, psychologists and patients was held in the Brandenburg Psychiatric Hospital, which became a biennial event.^93^ The self-published documentation of these conferences in Brandenburg is vital for this topic, offering insight into the various attempts or implementations of new approaches nationwide and, thus, into possible transnational transfer of knowledge.

## Western orientation and a new openness in the 1980s

Arriving in the 1980s, the domestic and international exchange further intensified through transnational cooperation and conferences, also visible in East German scientific journals. The bibliographies of articles included almost only references from the USA, Canada, Britain, and Scandinavia, with hardly any from Eastern Bloc states. The MfS registered these tendencies as a predisposed bias toward Western orientation, especially among the persistent bourgeois parts of the medical profession.^94^ Nevertheless, the state tolerated this exchange as long as it did not undermine the legitimacy and narrative of the GDR. Selected doctors from the GDR continued to participate in and report about the international conferences of the ICAA, and its twenty-third meeting was held in Dresden in 1977.^95^

Aside from these scientific conferences and open exchanges, state officials throughout the 1980s were reluctant to address alcohol addiction as an inherent social issue at the international and national levels. Although they could no longer use the ‘relic of capitalism’ paradigm, they continued to portray the problem as a development that was at odds with the socialist transformation.^96^ In this way, it remained a problem of the few who were distancing themselves from society, but not a problem of society. In the final section, I will explore how this dilemma between the recognition of the urgency of the issue and the unwillingness to change course, combined with the extended exchange across the Iron Curtain, led to fractures in GDR society.

According to an internal paper of the Ministry of Health, a copy of which has survived in the State Security Archive, in 1985, doctors from the Wilhelm-Griesinger Hospital in Berlin were commissioned to formulate lines of arguments for national and international events and proposals for tackling the problem of alcohol addiction and abuse. In their drafts, the unnamed authors described the current situation as follows:

The lack of information among experts and laypeople is glaring. It is expressed in the tolerance of alcohol consumption by the general public – often to the point of excess – and the simultaneous categorical rejection of alcoholics even by doctors.^97^

While this statement ignored the local initiatives described and the people who actively worked to improve the situation of their patients, the authors confirmed the observation that the general attitude towards alcohol and drinking culture in the GDR had deteriorated. In 1988, East Germany became the world champion in liquor consumption, which was accompanied by a drastic increase in cases of addiction, as the authors also emphasised.^98^ However, there were never any state-wide statistics; expert estimates ranged between 100,000 and one million cases.^99^ With this context, the authors further evaluated the GDR’s international commitment to improving the situation:

As a respected member of the World Health Organisation, the GDR participated at best passively in the elaboration of strategies to combat abuse and addiction.^100^

Considering that, for the GDR, international recognition and involvement became crucial after Honecker took over, this judgment confirms the continued assertion that alcoholism was not a problem of a socialist society. While books and official statements from the last decades of the GDR often stated that some people ‘even in our country’ were – in contradiction to societal conditions – addicted to alcohol, the causes were ascribed to their personality and milieu. In 1985, for example, another internal paper argued that alcohol addiction and abuse only existed due to ‘drinking customs that have not been overcome, traditional habits of thought and behaviour as remnants of the old social order’ and ‘inactive leisure time behaviour with passive psychological stimulation’.^101^ Even if the reasons cited for addiction could be valid, this statement shows that almost forty years after the founding of the GDR, the problem was still externalised by state functionaries as a relic of capitalism, a contradiction to socialism, and the fault of the individual. Due to the ideological struggle with the West, they continued to deny that alcohol addiction could also be a general social problem under socialism. The unnamed authors of the 1985 paper described the result of this situation as follows:

For research into the conditions and effects and prevention of alcohol abuse and alcohol dependence currently exists neither a research nor a combating strategy in the GDR.^102^

Many of the aspects mentioned by the authors resemble the criticism of Thränhardt from 1966 and reveal the persistent stagnation at the state level and in the Ministry of Health in particular. However, this renewed harsh internal criticism of the conditions in the GDR regarding alcohol addiction also fits the thesis that, on the one hand, the state officials delayed decisions, general strategies, and open statements. On the other hand, they indirectly tolerated solutions and methods from the West and relied on the initiatives and improvements in the periphery.

Change continued to take place in the local sphere. Special medical departments for people with addictions opened in the occupational health clinics in some companies across the country, like at the Neptun Wharf in Rostock.^103^ Doctors introduced the ‘Munich Alcoholism Test’ (MALT), developed by the West German doctor Wilhelm Feuerlein (1920–2015) and his team in 1979, to assess individual cases of alcohol addiction for therapeutic plans.^104^ East German reformers, like Windischmann, established more platforms for doctors, psychologists, and patients to meet and discuss their concepts for improving the treatment and aftercare of people with addictions, such as the Dresden Club Talks.^105^ Therapeutic clubs and self-aid organisations of patients, unthinkable in the 1960s, had spread across the country, and by 1988, Alcoholics Anonymous (AA) had established its first branch in East Germany.^106^ It appears that the experts and groups mentioned gained significant leeway for their actions due to their calculated defiance, offered solutions, and the state’s hesitation.

In Arnsdorf during the 1980s, doctors and psychologists also used the MALT, and the classification by Jellinek became predominant. Compared to previous decades, the therapeutic methods in the 1980s relied heavily on group and individual psychotherapy, targeted and purposeful work therapy, and more self-reflection exercises such as diary writing and consultations with a psychologist. The two ‘alcohol tests’ were still in use to familiarise patients with the effects of disulfiram, which they had to take regularly over a longer period, in line with the international standard.^107^ However, a further change occurred when Koritsch left the hospital in 1986 and ultimately East Germany in 1988. According to Hasso Engel (b. 1933), a psychologist and one of the organisers of the Dresden Club Talks, Koritsch’s departure resulted in a backlash in Arnsdorf. In a meeting at the ‘District Office for the Care of Alcohol and Pharmaceutical Addicts’ in 1987, Engel criticised the medical director of Arnsdorf, Wieder, who also participated, and the current state of treating people with alcohol addiction. In the opinion of Engel, it ‘can be compared to safekeeping rather than a proper and professional treatment’.^108^ This subjective observation by Engel, which was also based on an obvious antipathy towards Wieder, confirms that the introduction of reforms and Western methods and, thus, the experiences of people with alcohol addiction depended more on those in charge on-site and their attitudes than on ideological postulates by the state.^109^

## Conclusion

Over the last decade, studies have shown that the Iron Curtain was never insurmountable during the Cold War, especially for intangibles such as ideas and theories. Even for ideologically sensitive issues such as alcohol addiction or suicide rates, the state never implemented a complete ban on Western influences. Instead, the situation appears to be conflicted between ideological claims, pragmatism, and tolerance of expertise exchange in an attempt to facilitate improvements and, also importantly, perhaps win the hearts and minds of medical professionals to the cause of socialism. As a vital group for society, doctors always had a rather antagonistic relationship to the GDR and often resisted ideological indoctrination, which made them a recalcitrant group in the eyes of the East German government.^110^

This article traced some international debates and the transfer of knowledge in the case of treating people with alcohol addiction, using the example of the District Hospital Arnsdorf. With this micro-study, which is embedded in the national and international context, I illustrated how, over the forty years of the GDR’s existence, doctors, psychologists, patients and local authorities had gained more leeway to try out new approaches in treating alcohol addiction, many of which came from both the East and the West. Whereas in the mid-1960s, the GDR still rejected any form of self-aid organisation by patients as unnecessary in a socialist healthcare system^111^, doctors and psychologists had already begun establishing ‘therapeutic clubs’ by the end of this decade. In their approaches, they often followed the procedure of Skála from Prague but also included ideas from Western experts or the AA. However, these groups differed in one crucial aspect: they were led by doctors or psychologists. In this way, it could be argued that they were still integrated into the state healthcare system and remained under ‘social control’, which was an essential aspect of the GDR.

By the 1980s, however, the term ‘self-aid’ also appeared in scientific discussions and in the descriptions of these patient groups, not least because their usefulness beyond ideological claims had been realised. Therefore, the sole reliance on aversion therapy, deterrence, and moral accusations in the first decades shifted towards a mixture of more specialised and individualised treatments. As the example of Arnsdorf shows, this development depended heavily on locality and those in charge, and the most important reason for change was a new generation of doctors and psychologists who became involved at local and international levels. Furthermore, the increasing transnational exchange of expertise, the pressing social problem at home, and pressure from the local level forced the GDR state leadership to react and lift some sanctions. For example, they allowed television programmes and biographical or fictional literature that dealt with the subject of alcohol addiction and described the experiences of those affected in the socialist state.^112^ However, the state stuck to its narrative that alcohol addiction was a ‘relic of capitalism’ or ‘contrary to socialism’. It was not until August 1989 that the state authorities introduced a comprehensive strategy against alcohol addiction, which is indicative of their role up to this point. Although the end of the GDR was not foreseeable just a few months later, they remained passive observers at the national and international levels until the very end. Furthermore, many proposals of the 1989 regulation had already been implemented at the local level for three decades by the aforementioned reformers, such as Windischmann.^113^ State authorities were trapped in the ideological framework, delayed decisions, could not agree on general strategies for decades, avoided open statements, and ultimately tolerated solutions and methods from the West and relied on initiatives and improvements in the periphery. This tactic enabled them to continue denying that this problem was also inherent in a socialist society.

## Footnotes

1

Erik Winter , ‘Gedanken zur Notwendigkeit eines Betreuungsnetzes für Alkohol- und andere Suchtkranke und zur Ausbildung von Suchttherapeuten - Einstimmung in eine Diskussion über Notwendigkeiten und Möglichkeiten’, in Hugo von Keyserlingk et al. (eds), Diagnostik und Therapie Suchtkranker: Ergebnisse der 1. Arbeitstagung der Arbeitsgruppe ‘Suchtkrankheiten’ der Sektion der Gesellschaft für Psychiatrie und Neurologie der DDR, Wustrow 27.-30. Oktober 1987 (Berlin: VEB Verlag Volk und Gesundheit, 1988), 41 .

2 Deutsches Hygiene Museum Dresden [DHMD] 2016/134.7: Exhibition poster of German Hygiene Museum in Dresden, Der Alkohol – Freund oder Feind?, ca. 1957.

3 Bundesarchiv [BArch], DP 1/7110, Bl. 13: Parteivorstand SED an Herrn Staatssekretär Peschke, Ministerium für Arbeit und Gesundheitswesen, 7. Juli 1950.

4 For more information, see Kathleen Haack et al., ‘Vom “wesensfremden Konsum”– Zum Umgang mit der Alkoholproblematik in einem DDR-Großbetrieb’, PPmP - Psychotherapie · Psychosomatik · Medizinische Psychologie, 72, 12 (2022), 558–6334937096 10.1055/a-1704-8565; Anton Schrödter, ‘Die Erfassung, Betreuung und Versorgung alkoholabhängiger Bürger in der DDR am Beispiel Rostocks im Zeitraum von 1970 bis 1990 mit besonderem Fokus auf den betrieblichen Sektor’(unpublished Dissertation: Universität Rostock, 2022), 1, 12–5.

5 See, for example, Stadtarchiv [StA] Stralsund, Rep. 49, Nr. 499, unpag.: Ratssitzung vom 16. Mai 1957; 4a: Vorlage zur Änderung des Beschlusses über den Verkauf von Spirituosen in Kiosken.

6 BArch, DQ 1/27014, unpag.: Nicht geordnete Informationen, 25. April 1979.

7

Viola Balz , ‘“Nur eine Cognacbohne”. Alkohol im Spiegel der Gesundheitsfilme des Deutschen Hygiene Museums Dresden’, in Ekkehardt Kumbier (ed.), Psychiatrie in der DDR: Beiträge zur Geschichte, Band 2 (Berlin: be.bra, 2020), 251 ; Jenny Linek , Gesundheitsvorsorge in der DDR zwischen Propaganda und Praxis (Stuttgart: Franz Steiner, 2016), 134 .

8

Thomas Kochan , Blauer Würger: So trank die DDR (Berlin: aufbau, 2011), 12 ; *Statistisches Jahrbuch der Deutschen Demokratischen Republik, 1989* (Berlin: Staatsverlag der Deutschen Demokratischen Republik, 1990), 292, online: <https://www.digizeitschriften.de/dms/toc/?PID=PPN514402644_1989>.

9 Kochan, *op. cit.* (note 8), 147–80.

10 BArch, DP 1/2485, unpag.: Beratungen über die Erfahrungen seit dem Inkrafttreten des StGB im Jahre 1968, 26. November 1969, p. 26.

11

Stefan Orlob , ‘Alkoholismus und Kriminalität in der DDR unter besonderer Berücksichtigung Jugendlicher und Heranwachsender’, in Ekkehardt Kumbier and Kathleen Haack (eds), Psychiatrie in der DDR III: Weitere Beiträge zur Geschichte (Berlin: BeBra Wissenschaft, 2023), 344 .

12 BArch, DQ 1/26453, unpag.: Betr.: Anordnung über die Behandlung und Ausnüchterung von Personen im Zustande der Trunkenheit, 02. Oktober 1961.

13 BArch, MfS, ZOS 3852, Bl. 60: Ausschnitt *Berliner Zeitung*, Mißbrauch wird teuer, 28. Dezember 1984.

14

Ingrid Johannis , Das siebente Brennesselhemd. Aus dem Tagebuch einer Alkoholkranken (Berlin: Neues Leben, 1986). For an overview of health films about alcohol abuse in the GDR, see Balz, *op. cit.* (note 7).

15

Michael Geyer (ed.), Psychotherapie in Ostdeutschland: Geschichte und Geschichten 1945-1995 (Göttingen: Vandenhoeck & Ruprecht, 2011).

16

Thomas Metan and Boris Böhm , 100 Jahre Krankenhaus Arnsdorf: Von der Königlich Sächsischen Pflegeanstalt zum Fachkrankenhaus für Psychiatrie, Psychotherapie und Neurologie (Dessau-Roßlau: HOTEX Verlag Thomas Metan, 2012), 75 .

17 Geyer, *op. cit.* (note 15), 87–95.

18 Doreen Scholtz and Holger Steinberg, ‘Die Theorie und Praxis der Pawlow’schen Schlaftherapie in der DDR’, *Psychiatrische Praxis*, 38, 07 (2011), 323–8.

19

Matthias Hoheisel , ‘Von der Suchtdroge zum Therapeutikum – Zur Bewertung von Beruhigungsmitteln an der Charité Nervenklinik von 1962–75’, Medizinhistorisches Journal, 47, 1 (2012), 74 23691877.

20

D. Müller-Hegemann , ‘Zur soziologischen Problematik der Suchten’, Psychiatrie, Neurologie und medizinische Psychologie, 3, 9 (1951), 268–7314900274 .

21

Helmuth Grage , ‘Über die Provokation von Psychosen bei der medikamentösen Behandlung des chron. Alkoholismus (Antabuspsychosen)’, Psychiatrie, Neurologie und medizinische Psychologie, 4, 4 (1952), 108–1114949243 ; F. Ruck , ‘Alkoholentziehungskur mit Hilfe eines bedingten Reflexes (Apomorphinentziehungskur)’, Psychiatrie, Neurologie und medizinische Psychologie, 8, 2/3 (1956), 88–92 13323135 .

22 For more information, see Markus Wahl , ‘“Ich weiß, daß die Kur sehr hart wird…”: Die Anwendung der Aversionstherapie bei Patienten mit Alkoholabhängigkeit im Bezirkskrankenhaus für Neurologie und Psychiatrie in Arnsdorf in der DDR, 1966-1981’, in Ekkehardt Kumbier (ed.), Psychiatrie in der DDR: Beiträge zur Geschichte, Band 2 (Berlin: be.bra, 2020), 229–42.

23

Kate Davison , ‘Cold War Pavlov: Homosexual Aversion Therapy in the 1960s’, History of the Human Sciences, 34, 1 (2021), 89–119 .

24

Dieter Hörig , ‘Alkohol und Geschlechtskrankheiten’, Alles für Deine Gesundheit - Deutsches Hygiene Museum Dresden, 2 (1959), 7 ; Karl Welich , ‘Alkohol und Jugendkriminalität’, Alles für Deine Gesundheit - Deutsches Hygiene Museum Dresden, 2 (1959), 6 .

25

Klaus Vogel and Peter Bexte , Das Deutsche Hygiene-Museum Dresden 1911 bis 1990, Deutsches Hygiene-Museum and Stiftung Deutsches Hygiene-Museum (eds) (Dresden: Michel Sandstein Verlag, 2003).

26 Balz, *op. cit.* (note 7); Rudolf Neubert , Jugend und Alkohol, 2nd edn (Rudolstadt: Greifenverlag, 1959); ‘Der Alkohol - dein Freind! Der denkende Arbeiter trinkt nicht - der trinkende Arbeiter denkt nicht’, *Alles für Deine Gesundheit - Deutsches Hygiene Museum Dresden*, 3 (1960), 6.

27 For example, the consumption per capita of beer increased from 19.6 l to 79.5 l between 1950 and 1960. BArch, DQ 1/27014, unpag.: Aus der Klinik für Alkohol- und Drogenkranke (Chefarzt: Dr. E. Winter) des Wilhelm-Griesinger-Krankenhauses (Ärztlicher Direktor: Doz. Dr. sc. B. Nickel), Berlin-Biesdorf. Hinweise zu Alkoholgebrauch, Alkoholmißbrauch und Alkoholabhängigkeit in der Deutschen Demokratischen Republik. Von Erik Winter, April 1979, p. 3.

28 See, for example, BArch, DQ 1/21817, unpag.: Verordnung über die Unterbringung psychisch Kranker, psychisch Abwegiger und Süchtiger in Krankenanstalten und in Pflegeeinrichtungen [1956]; Verordnung über die vorübergehende Unterbringung von Personen im Zustand der Trunkenheit und Maßnahmen zur Bekämpfung des Alkoholmißbrauchs [1958].

29 BArch, DQ 1/23059, unpag.: Gesundheitsminister Max Sefrin an den Magistrat von Groß-Berlin, Herrn Stadtrat Rechner. Entwurf der Verordnung zur Bekämpfung des Alkoholismus, 23. Mai 1959.

30 See, for example, H. v. Keyserlingk, ‘Pervitin’, *Psychiatrie, Neurologie Und Medizinische Psychologie*, 2, 1 (1950), 1–9; H. v. Keyserlingk, ‘Soziologie, Behandlung Und Rehabilitation Der Alkoholiker’, *Psychiatrie, Neurologie Und Medizinische Psychologie*, 12, 5 (1960), 176–83; H. v. Keyserlingk, ‘Die Suizidgefährdung Der Alkoholiker’, *Psychiatrie, Neurologie Und Medizinische Psychologie*, 12, 4 (1960), 123–5; H. v. Keyserlingk, ‘Über Den Alkoholismus Bei Frauen’, *Psychiatrie, Neurologie Und Medizinische Psychologie*, 14, 7 (1962), 268–72.

31 BArch, DQ 1/21817, unpag.: Betr.: Gründung eines “Nationalen Komitees zum Studium des Alkoholismus in der DDR”, 27. Juni 1958.

32 Robin Room, ‘Archer Tongue, 1919–2006: Building an International Forum for the Addictions Field’, *Addiction*, 102, 4 (2007), 668.

33 BArch, DP 1/3851, Bl. 102: 31. Hauptabteilung III – Sektor Strafrecht an Abteilung 1: Internationaler Kongreß über Alkoholismus und Drogenabhängigkeit vom 23. – 28.2.1975 in Bangkok (Thailand), Oktober 1974.

34 BArch, DQ 1/21817, unpag.: Zweiter Sommerkurs zum wissenschaftlichen Studium der Verhütung des Alkoholismus, 25. Juni – 6. Juli 1956, Genf – Schweiz, Schutz der Jugend vor dem Alkohol von Professor Dr. med. R. Neubert, Jena.

35 For example, even in internal reports of the Ministry of State Security in 1985. BArch, MfS, HA XX 1901, Bl. 2: Information über ausgewählte sozial- und gesundheitspolitische Aktivitäten der DDR gegen Alkohol sowie Sucht- und Arzneimittelmißbrauch, 22. März 1985.

36

Christian Sammer , ‘Vom Aufstieg und Fall der Utopie Gesundheit: Konzepte, Strukturen und Grenzen der Gesundheitsaufklärung im sozialistischen Gesundheitswesen der DDR’, in Markus Wahl (ed.), Volkseigene Gesundheit : Reflexionen zur Sozialgeschichte des Gesundheitswesens der DDR (Stuttgart: Franz Steiner, 2020), 179–90; Christian Sammer , ‘Gesundheitserziehung – die Ver(sozial)wissenschaftlichung der Gesundheitsaufklärung in den 1950er und 1960er Jahren’, NTM - Zeitschrift für Geschichte der Wissenschaften, Technik und Medizin, 27, 1 (2019), 179–9710.1007/s00048-018-00203-630666339.

37 BArch, DQ 1/23059, unpag.: Bericht über das 12. Internationale Seminar zur Verhütung und Behandlung des Alkoholismus, p. 9.

38 BArch, DQ 1/26453, unpag.: Betr.: Anordnung über die Behandlung und Ausnüchterung von Personen im Zustande der Trunkenheit, 02. Oktober 1961.

39 BArch, DQ 1/23059, unpag.: Bericht über das 12. Internationale Seminar zur Verhütung und Behandlung des Alkoholismus, p. 9.

40

Elvin Morton Jellinek , The Disease Concept of Alcoholism, 7th edn (New Haven: College and University Press, 1983).

41 BArch, DQ 1/23059, unpag.: Bericht über das 12. Internationale Seminar zur Verhütung und Behandlung des Alkoholismus, p. 3.

42 See, for example, the chapters on different countries and their application of aversion therapy and disulfiram in Waltraud Ernst and Thomas Müller (eds), Alcohol, Psychiatry and Society: Comparative and Transnational Perspectives, c. 1700–1990s, Vol. 51 (Manchester: Manchester University Press, 2022).

43 BArch, DQ 1/23059, unpag.: Bericht über das 12. Internationale Seminar zur Verhütung und Behandlung des Alkoholismus, p. 4.

44

Adéla Gjuričová , ‘A Cradle of Psychotherapy: Treatment of Alcohol Addiction in Communist Czechoslovakia, c. 1948–1989’, in Waltraud Ernst and Thomas Müller (eds), Alcohol, Psychiatry and Society: Comparative and Transnational Perspectives, c. 1700–1990s, Vol. 51 (Manchester: Manchester University Press, 2022), 331–53; Esther Wahlen , ‘Turning Women into Alcoholics: The Politics of Alcohol in Late Socialist Czechoslovakia’, in Heike Karge et al. (eds), From the Midwife’s Bag to the Patient’s File: Public Health in Eastern and Southeastern Europe (Budapest: Central European University Press, 2017), 243–62.

45 Gjuričová, *op. cit.* (note 42), 347.

46

Ehrig Lange , ‘Bericht über die wissenschaftliche Arbeitstagung zu Grundsatzfragen der Behandlung Alkoholkranker am 26. Juni 1968 im Bezirkskrankenhaus für Psychiatrie und Neurologie Arnsdorf bei Dresden’, Psychiatrie, Neurologie und medizinische Psychologie, 21, 10 (1969), 394–6.

47 Clara Susanne Wiegmann, ‘Die Diskussion über die Ausgestaltung der DDR-Psychiatrie anhand ostdeutscher medizinischer Fachzeitschriften 1946–1989’(unpublished Dr. med.: Eberhard Karls Universität zu Tübingen, 2018), 85.

48 Lange, *op. cit.* (note 44), 394.

49 ‘Neues Opfer im “Malaria-Skandal”: Die Vorwürfe gegen die Wiener Psychiatrie reißen nicht ab. Verhaltensauffällige Kinder wurden als “irre” abgestempelt’, *Kurier*, 8 February 2012, online: <https://kurier.at/chronik/wien/neues-opfer-im-malaria-skandal/767.458>, accessed 7 May 2024; ‘“Malariatherapie”: Anwalt: “Opfermeldungen reißen nicht ab”. Bisher rund zehn Personen in Kontakt mit Wiener Juristen’, *Der Standard*, 10 February 2012, online: <https://www.derstandard.at/story/1328507430104/malariatherapie-anwalt-opfermeldungen-reissen-nicht-ab>, accessed 7 May 2024.

50

Kornelius Kryspin-Exner , Die offene Anstalt für Alkoholkranke in Wien-Kalksburg: Methoden und Ergebnisse, 1961-1966 (Hollinek, 1967).

51 Lange, *op. cit.* (note 44), 394.

52
*Ibid.*, 394–5.

53
*Ibid.*, 395.

54 See, for example, Sächsisches Krankenhaus Arnsdorf [SKHA], Medizinisches Archiv, Nr. 222, unpag.; SKHA, Medizinisches Archiv, Nr. 226, unpag.; SKHA, Medizinisches Archiv, Nr. 228, unpag.

55

Hans-Joachim Wenzel and Hubertus Windischmann (eds), Dokumentation: Erfahrungsaustausch abstinent lebender Alkohol- und Drogenkranker in der DDR, Bezirksnervenklinik Brandenburg 4. u. 5.5.79 (Brandenburg: Bezirksnervenklinik Brandenburg, 1979), 65–8.

56

Sigurd Kasischke , Die Geschichte der AGAS: Evangelische Arbeitsgemeinschaft zur Abwehr der Suchtgefahren (Lüdenscheid: Blaukreuz-Verlag, 2013).

57 SKHA, Medizinisches Archiv, Nr. 791/68, unpag.

58 Name anonymised.

59 SKHA, Medizinisches Archiv, Nr. 791/68, unpag.

60

Emmanuel Droit , Vorwärts zum neuen Menschen? Die sozialistische Erziehung in der DDR (1949 - 1989), Michael Esch (trans.) (Cologne: Böhlau, 2013); Verena Zimmermann , ‘Den neuen Menschen schaffen’: Die Umerziehung von schwererziehbaren und straffälligen Jugendlichen in der DDR (1945–1990) (Cologne: Böhlau, 2004).

61 For the issue of socialisation of doctors and its impact on the treatment of stigmatised diseases, see Markus Wahl , ‘Treatments from the Past: Continuities in Treating Venereal Diseases’, Medical Memories and Experiences in Postwar East Germany: Treatments of the Past (London: Routledge, 2019), 72–116 .

62 For more details, see Wahl, ‘“Ich weiß, daß die Kur sehr hart wird…”’, *op. cit.* (note 21).

63 See, for example, SKHA, Medizinisches Archiv, Nr. 791/68, unpag.; SKHA, Medizinisches Archiv, Nr. 2134/68, unpag.; SKHA, Medizinisches Archiv, Nr. 150/68, unpag.

64 Lange, *op. cit.* (note 44), 395.

65 SKHA, Medizinisches Archiv, Nr. 791/68, unpag.

66

Markus Wahl , ‘Der Patient im Betrieb: Erfahrungen von diabetes- und alkoholkranken Menschen am Arbeitsplatz in der DDR’, in Markus Wahl (ed.), Volkseigene Gesundheit: Reflexionen zur Sozialgeschichte des Gesundheitswesens der DDR (Stuttgart: Franz Steiner, 2020), 71–92 .

67 Lange, *op. cit.* (note 44), 395.

68 SKHA, Medizinisches Archiv, Nr. 357/71, unpag.

69 Metan and Böhm, *op. cit.* (note 16), 72.

70 Interview with Hubertus Windischmann, 25 November 2019.

71 BArch, DQ 1/23059, unpag.: Bericht über das 12. Internationale Seminar zur Verhütung und Behandlung des Alkoholismus, p. 12.

72

Monika Storch et al., ‘Psychotherapeutische Fachliteratur der DDR und BRD: Eine vergleichende Zitationsanalyse’, PPmP - Psychotherapie · Psychosomatik · Medizinische Psychologie, 72, 07 (2022), 316–2435114715 10.1055/a-1718-4071.

73

Ekkehardt Kumbier et al., ‘50 Jahre Rodewischer Thesen – Zu den Anfängen sozialpsychiatrischer Reformen in der DDR’, Psychiatrische Praxis, 40 (2013), 313–2023868717 10.1055/s-0033-1343229.

74 BArch, MfS, HA XX 479, Bl. 393: Ärzte gegen die Einweisung von Patienten anlässlich politischer Höhepunkte, 1973.

75

Sonja Süß , Politisch mißbraucht? Psychiatrie und Staatssicherheit in der DDR, 2nd edn (Berlin: Links, 1999), 542–53.

76 For the general situation in the healthcare system of the GDR, see Rainer Erices and Antje Gumz , ‘“Hier läuft bald gar nichts mehr”. BStU-Quellen zur Entwicklung des Gesundheitswesens in der DDR’, in Andreas Frewer and Rainer Erices (eds), Medizinethik in der DDR: Moralische und menschenrechtliche Fragen im Gesundheitswesen (Stuttgart: Franz Steiner, 2015), 15–28 .

77

Siegfried Schirmer , ‘Handhabung des Einweisungsrechts’, Das Deutsche Gesundheitswesen, 27 (1972), 855–85037376 ; ‘Gesetz über die Einweisung in stationäre Einrichtungen für psychisch Kranke vom 11. Juni 1968’, *Gesetzesblatt der Deutschen Demokratischen Republik*, Teil I, 13 (1968), 273–6.

78

Henriette Voelker , ‘Therapeutisches Schreiben in der Charité: Alltagspraxis und Subjektivierung in der Gruppenpsychotherapie, 1974–1978’, Medizinhistorisches Journal, 58, 3 (2023), 191–223 .

79

Harald Rogoll and Helmuth F. Späte , ‘Soziale und medizinische Charakteristika kreisärztlich eingewiesener psychisch Kranker’, Psychiatrie, Neurologie und medizinische Psychologie, 36, 3 (1984), 146–516728964 .

80

Helmut F. Späte and Harald Rogoll , ‘Normative und verfahrensrechtliche Voraussetzungen bei der Einweisung psychisch Kranker gemäß § 6 des Einweisungsgesetzes der DDR’, Psychiatrie, Neurologie und medizinische Psychologie, 36, 8 (1984), 489–956494344 .

81

Siegfried Schirmer et al., ‘Neun Thesen zur Therapeutischen Gemeinschaft (zugleich Diskussionsgrundlage für die am 2./3. Mai 1974 in der Bezirksnervenklinik Brandenburg stattfindenden Tagung zu diesem Thema)’, Psychiatrie, Neurologie und medizinische Psychologie, 26, 1 (1974), 50–4; Siegfried Schirmer et al., ‘Brandenburger Thesen zur Therapeutischen Gemeinschaft’, Psychiatrie, Neurologie und medizinische Psychologie, 28, 1 (1976), 21–5940880 .

82

Ekkehardt Kumbier and Kathleen Haack , ‘Die Therapeutische Gemeinschaft und das Arzt-Schwester-Patient-Verhältnis in der Psychiatrie: Zwischen therapeutischen Anspruch und sozialistischer Realität’, in Markus Wahl (ed.), Volkseigene Gesundheit: Reflexionen zur Sozialgeschichte des Gesundheitswesens der DDR (Franz Steiner Verlag, 2020), 114–20.

83 Schirmer et al., ‘Neun Thesen’, *op. cit.* (note 79); Schirmer et al., ‘Brandenburger Thesen’, *op. cit.* (note 79).

84 SKHA, Medizinisches Archiv, Nr. 940/75.

85 See for example, SKHA, Medizinisches Archiv, Nr. 1005/75, unpag.; SKHA, Medizinisches Archiv, Nr. 1288/75, unpag.; SKHA, Medizinisches Archiv, Nr. 1009/75, unpag.

86 See, for example, SKHA, Medizinisches Archiv, Nr. 359/71, unpag.

87 See, for example, Sächsisches Hauptstaatsarchiv Dresden [SächsStA-D], 10820 Landesheil- und Pflegeanstalt /Bezirkskrankenhaus für Neurologie und Psychiatrie Arnsdorf, Nr. 955, unpag.

88 Wahl, ‘“Ich weiß, daß die Kur sehr hart wird…”: Die Anwendung der Aversionstherapie bei Patienten mit Alkoholabhängigkeit im Bezirkskrankenhaus für Neurologie und Psychiatrie in Arnsdorf in der DDR, 1966-1981’, *op. cit.* (note 21); Markus Wahl , ‘Doing Drugs in Socialist East Germany: Gendered Prescription and (Ab-)Use of Pharmaceuticals in the GDR, 1949–1989’, Social History of Alcohol and Drugs, 35, 2 (2021), 287–326 .

89 See, for example, SächsStA-D, 10820 Landesheil- und Pflegeanstalt /Bezirkskrankenhaus für Neurologie und Psychiatrie Arnsdorf, Nr. 1037, unpag.; SKHA, Medizinisches Archiv, Nr. 06266, unpag.

90

Hans-Dieter Koritsch , ‘Die Rolle des Disulfiram in der Entwöhnungsbehandlung Alkoholabhängiger’, Zeitschrift für ärztliche Fortbildung, 76 (1982), 454–77136031 .

91 Geyer, *op. cit.* (note 15), 90, 144, 243.

92 Wahl, ‘“Ich weiß, daß die Kur sehr hart wird…”’, *op. cit.* (note 21), 238–42.

93

H. K. Kursawe , ‘Über den ersten Erfahrungsaustausch abstinent lebender Alkoholiker am 5. und 6. Mai 1979 in der Bezirksnervenklinik Brandenburg (Ärztlicher Direktor: MR Dipl.-Jur. Dr. med. S. Schirmer)’, Psychiatrie, Neurologie und medizinische Psychologie, 32, 3 (1980), 181–181 ; Wenzel and Windischmann, *op. cit.* (note 53).

94 BArch, MfS, HA XX, 1470, Bl. 105–8: Informationen über die Zustände im Gesundheitswesen [Bezirk Dresden], 1987.

95

E. J. Tongue and I. Moos (eds), 23. Internationales Seminar zur Verhütung und Behandlung des Alkoholismus, Dresden, 6.6.-10.6.1977 (Lausanne: International Council on Alcohol and Addictions, 1977).

96 Orlob, *op. cit.* (note 11), 337. See also BArch, MfS, HA XX, 1901, Bl. 2–12: Information über ausgewählte sozial- und gesundheitspolitische Aktivitäten der DDR gegen Alkohol sowie Sucht- und Arzneimittelmißbrauch, 22. März 1985.

97 BArch, MfS, HA XX, 1901, Bl. 24: Die gesundheitspolitische Bedeutung von Alkoholmißbrauch und Alkoholabhängigkeit in der DDR unter besonderer Berücksichtigung volkswirtschaftlicher Aspekte, [without date], p. 2.

98
*Ibid.*, Bl. 25.

99

Hubertus Windischmann , Ein Gläschen in Ehren… (Berlin: VEB Verlag Volk und Gesundheit, 1989), 70 .

100 BArch, MfS, HA XX, 1901, Bl. 24: Die gesundheitspolitische Bedeutung von Alkoholmißbrauch und Alkoholabhängigkeit in der DDR unter besonderer Berücksichtigung volkswirtschaftlicher Aspekte, [without date], p. 2.

101 BArch, MfS, HA XX, 1901, Bl. 3: Information über ausgewählte sozial- und gesundheitspolitische Aktivitäten der DDR gegen Alkohol sowie Sucht- und Arzneimittelmißbrauch, 22. März 1985.

102 BArch, MfS, HA XX, 1901, Bl. 24: Die gesundheitspolitische Bedeutung von Alkoholmißbrauch und Alkoholabhängigkeit in der DDR unter besonderer Berücksichtigung volkswirtschaftlicher Aspekte, [without date], p. 2.

103 See especially chapter 7 of Schrödter, *op. cit.* (note 4).

104

Wilhelm Feuerlein , Alkoholismus: Warnsignale - Vorbeugung - Therapie, 6th edn (München: Beck, 2008); Wilhelm Feuerlein et al., Sucht und Mann: Ansätze, Argumentationen, Ausblicke (Hamm: Hoheneck, 1988).

105 See, for example, Hasso Engel (ed.), ‘Probleme und Ergebnisse der Psychotherapie bei Aloholkranken’: Dokumentation. Wissenschaftliche Hauptveranstaltung des 8. Dresdner Klubgespräches (Dresden, 1986); Hasso Engel (ed.), Der Alkoholkranke in der Sprechstune des Haus- und Betriebsarztes: Dokumentation der Wissenschafltichen Hauptveranstaltung des 9. Dresdner Klubgespräches (Dresden, 1987).

106

Hubertus Windischmann , ‘Zur Geschichte der Abstinenzbewegung in der DDR’, in Reinhold Beß et al. (eds), Therapeutische Gemeinschaften für süchtige Menschen in Europa: Referate der Europäischen Konferenz der Therapeutischen Gemeinschaften, Berlin 6.-9. Juni 1990 (München: Röttger, 1991), 91–101 .

107 See, for example, SKHA, Medizinisches Archiv, Nr. 20350, unpag.

108 SächsStA-D, 10822 Landesanstalt/Fachkrankenhaus Großschweidnitz, Nr. 12048, unpag.: Protokoll, Kreisstelle für die Betreuung Alkohol- und Arzneimittelabhängiger, Rat der Stadt Dresden, 05. Februar 1987.

109 For more information, see Markus Wahl , ‘Treating East German Subjects: Doctors and Patients with Alcohol Addiction in Late Socialism’, in Scott Harrison et al. (eds), Socialist Subjectivities: Queering East Germany under Honecker (Ann Arbor, M.I.: University of Michigan Press, 2024), 58–82 .

110 For the struggle between the state and the medical profession throughout the GDR’s existence, see Anna-Sabine Ernst , ‘Die beste Prophylaxe ist der Sozialismus’: Ärzte und Hochschullehrer in der SBZ/DDR 1945–1961 (Münster: Waxmann, 1996); Markus Wahl, ‘“It Would Be Better, If Some Doctors Were Sent to Work in the Coal Mines”: The SED and the Medical Intelligentsia Between 1961 and 1981’(unpublished Master’s thesis: University of Canterbury, 2013); Markus Wahl , ‘“Warum habt ihr solche Angst, dass wir nicht wiederkommen?” ‘Grenzübertritte’ der medizinischen Intelligenz in den 1970er Jahren’, in Andreas Frewer (ed.), Medizinethik in der DDR: Moralische und menschenrechtliche Fragen im Gesundheitswesen (Stuttgart: Franz Steiner, 2015), 59–80 .

111 See, for example, BArch, DQ 1/4346, unpag.: Brief, Abt. Organisation des Gesundheitsschutzes – spezielle medizinische Betreuung an Herrn W. M., 23. Juni 1967.

112 Johannis, *op. cit.* (note 14).

113 Kochan, *op. cit.* (note 8), 313–4.

